# Critical Overview of Patriarchy, Its Interferences With Psychological Development, and Risks for Mental Health

**DOI:** 10.7759/cureus.40216

**Published:** 2023-06-10

**Authors:** Mayank Gupta, Jayakrishna S Madabushi, Nihit Gupta

**Affiliations:** 1 Psychiatry and Behavioral Sciences, Southwood Psychiatric Hospital, Pittsburgh, USA; 2 Psychiatry, Alabama College of Osteopathic Medicine, Birmingham, USA; 3 Psychiatry, Dayton Children’s Hospital, Dayton, USA

**Keywords:** gender discrimination, trauma, child developmental, feminism, mental health, patriarchy

## Abstract

The systemic oppression of women and gender-based discrimination has deep roots in human civilization. As evident in both written texts and widespread practices, conscious and unconscious biases associated with patriarchy have been and continue to be interlaced with power struggles, control, and conformity enforced by the male-dominant cultures of the time. Brought into bold relief in this pandemic, recent dramatic events (the tragic murder of George Floyd and the overturning of Roe v. Wade, for example) have heightened social outrage against bias, racism, and bigotry and have also brought us to an inflection point demanding our better understanding of the pernicious and long-term mental health effects of patriarchy. There are compelling grounds to further expand their construct, but efforts to do so in psychiatric phenomenology have, until now, failed to gain momentum and substantive attention. The resistance may in part lie in misconceptions that patriarchy is supported by archetypal endowments of the collective unconscious constitutive of shared societal beliefs. While many continue to live with the adverse experiences associated with patriarchy within the current times, critics have argued that our concepts about patriarchy are not empirical enough. Empirically supported deconstruction is necessary to debunk misinformed notions that undermine women’s equality.

## Introduction and background

In the last few decades, transformational technological advances have rapidly ushered in newer human behaviors, and we have witnessed paradigmatic shifts in the socio-cultural landscape of human civilizations. Renaissance and empiricism movements laid the foundation for advances in modern scientific methodology that challenged centuries of dogmatism. Technologies deriving from scientific inquiries outpace millions of years of gradual change. Yet the quest to infer the unmanifested mind remains a challenge even as mental health's significance is increasingly acknowledged in news, social media, journals, and everyday conversation.

Since 2020 there have been some critical turns of events. First, the widespread misinformation rejecting the existence of the SARS-COV-2 pandemic, and then the tragic murder of George Floyd that led to a hyperpolarized society and civil unrest [1]. Lastly, there was outrage about the U.S. Supreme Court's decision in Dobbs v. Jackson Women's Health Organization, effectively overturning Roe v. Wade, thereby restricting an individual's right to abortion [2,3]. Considering these events, the surge in the epidemiological trends of mental disorders without access to healthcare highlights the imminent status quo and inspires the need for alternative ideas. Amidst these developments, in 2021 a declaration of a National State of Emergency in Children’s Mental Health came from the American Academy of Paediatrics, American Academy of Child and Adolescent Psychiatry, and Children’s Hospital Association. Complex, intertwined, and confounding multifactorial aetiologies are gaining more recognition [4,5]. There are organizational efforts to develop systematic inquiries and uncover potential blind spots by applying the principles of scientific skepticism to psychiatric phenomenology [6]. The overarching pledge is to create a multifaceted understanding of the transgenerational effects of race, racism, social justice, and equity. There is a compelling rationale to expand the scope of the examination to include the societal institution of patriarchy, its marked pervasiveness in individual and social existence, and its pivotal role in human development. There is, indeed, an urgent need for systematic scientific verification of the relationship between gender-based discrimination issued from patriarchal worldviews and mental health trajectories for children, adolescents, and youths. However, without first having a coherent understanding of the essential construct that might command substantial consensus among stakeholders and, in turn, lead to objective measures to assess and refine it, there will remain a steep gap in the clinical practices of contextual psychiatry.

A panoramic overview of the rich literature scattered across disciplines is provided to establish the groundwork for a better understanding of the relationship of patriarchy to the psyche. Given the systemic omnipresence of patriarchy and the likelihood of subtle indoctrination among children and youths, this overview has implications in the context of both developmental psychopathology and implementing measures for course correction. 

This article was previously posted to the www.researchsquare.com preprint server on January 09, 2023.

## Review

Methods

**Figure 1:**
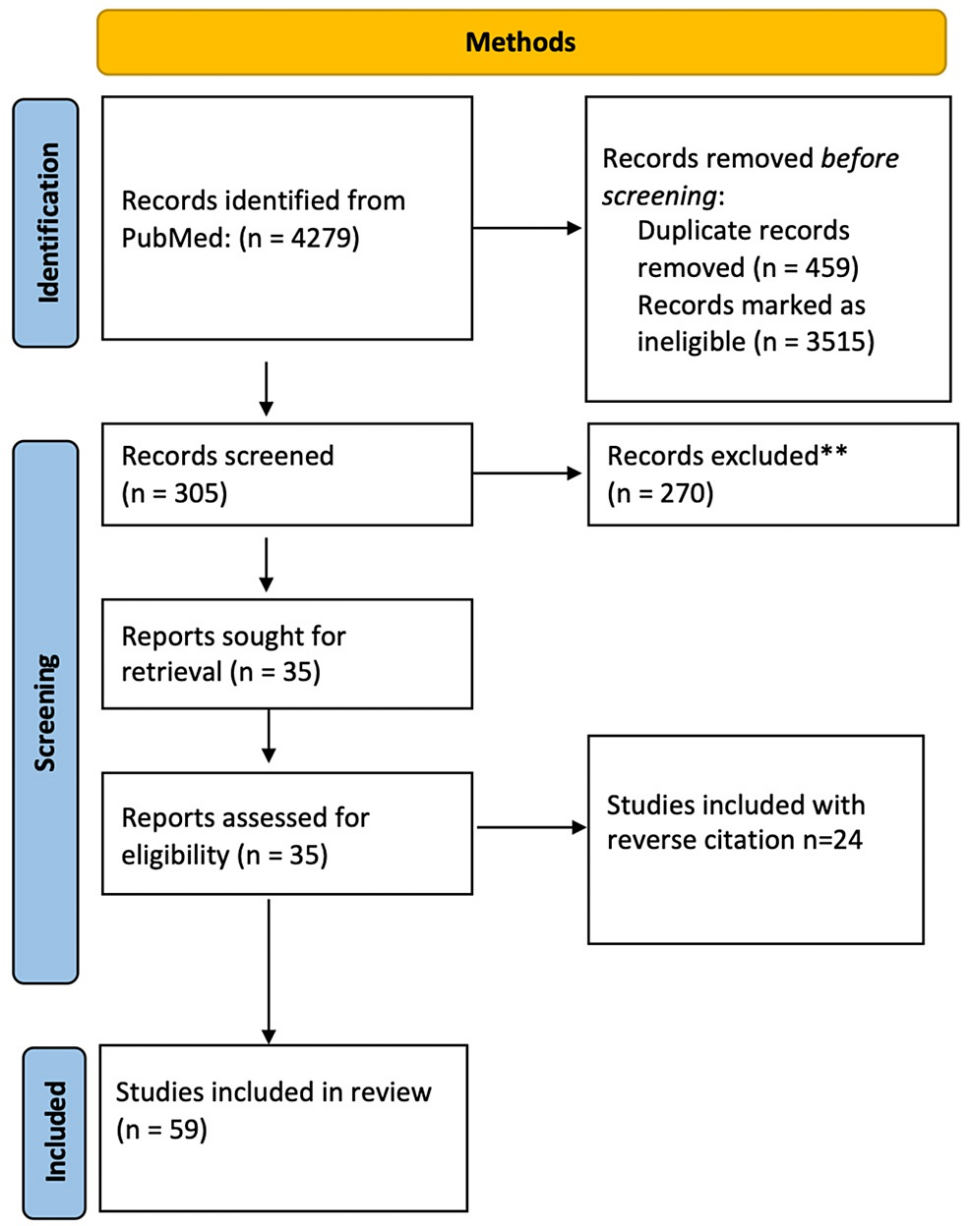
Summary of the search strategy **270 articles were removed as they were not relevant to the topics of patriarchy and mental health

A comprehensive search of several databases from the date of inception to the date of the search was conducted. The databases include PubMed, PsychINFO, Cochrane Library, and Google Scholar. We also searched the database of ongoing clinical trials through clinicaltrials.gov. The search was designed using controlled vocabulary and keywords such as "Patriarchy*", "Mental Health"," Feminism", "Trauma*", "Adverse Childhood Experiences", "Anthropology", "Developmental Psychopathology", Gender Discrimination Therapy,", and "Social Determinant*". It was performed in all languages and was limited to human subjects. We also performed a manual search. The inclusion criteria were any published material on patriarchy across all ages with links to mental health. Studies focused on social determinants associated with gender-based discrimination, patriarchy, and developmental psychopathology were selected for the review. We identified 305 published materials after the removal of duplicates. After reviewing the abstract, only 35 studies met our inclusion criteria. And 24 other studies were added manually after reverse citations were reviewed to update the material. Figure 1 provides the details.

Is patriarchy a coherent concept?

Patriarchy is often used loosely to indicate women’s oppression through male domination. There is a growing body of literature studying the impact of patriarchy (as a social determinant) on psychological functioning, and there are numerous theoretical explanations of patriarchy stemming from various fields, including not only the social and political sciences but also the humanities [7]. A contextual understanding of patriarchy’s deeply entrenched roots would be incomplete without diving into historical literature. Feminist movements paved the way for systematic studies of patriarchy; both Anglo-American and French feminist criticism and theory, for example, offer unique insights into the term "patriarchy." The former meditated on the concept of gender in a patriarchal society, and the latter did so in the specific context of literature and art [8]. In his sociological definition of patriarchy as a system of government in which men rule societies through their positions at the head of their families, Max Weber refers to "Herrschaft" a relationship based on the domination of men over women and subordinate men in households [9]. Critics have found this definition to be focused too exclusively on domination and submission and consequently grossly lacking in intersectionality.

Given the complexity of the topic, it is imperative to examine the evolution, perpetuation, and factors associated with the persistence of patriarchy. A panoramic overview of its mention in the various forms of literature provides insight from various vantage points.

Apologists

Among Western cultures, there are accounts from early Celtic settlements (6th to 11th centuries) in which women were legally equal to men [10]. They could hold and sell property, marry or divorce, and hold high educational degrees (physician, lawyer, or religious). Subsequently, Gaelic Christianity was brought into alignment with Roman Catholic orthodoxy [11]. ‘Traditional’ patriarchal concepts of marriage, equality, authority, and ordination became dominant in biblical interpretation and canonical literature [12]. That priestly ordination can only be conferred upon men is controversial to this day.

Although scripture states God created men and women as equals in his image, giving them both dominion over the earth and all living things, the writings of noted theologians declare otherwise. St. Thomas Aquinas (Summa Theologica, q. 92 a.) writes: "Woman is defective and misbegotten, for the active force in the male seed tends to the production of a perfect likeness in the masculine sex; while the production of a woman comes from defect in the active force or some material indisposition…" [13].

St. Augustine (De Genesi ad litteram, 9, 5-9 ) writes: "I don’t see what sort of help woman was created to provide man with if one excludes the purpose of procreation. If a woman was not given to a man for help in bearing children, for what help could she be…?" These writings influenced the development of Western philosophy and Western Christianity.

Likewise, in Eastern theology, the ancient Sanskrit scripture Manusmṛiti dating back from the 2nd century BCE to the 3rd century CE was a systematic legal text of written codes establishing Brahminic (caste-based social order) patriarchy. The text discusses denying women the right to bodily integrity, marriage rights, contraception, divorce, etc. [14]. Women are objectified and considered sexually promiscuous in a deeply disturbing misogynist set of rules (dharma) in which subordination is celebrated to propagate the patriarchy as natural.

In the rules of medieval common law in England, widows were usually automatically entitled to a third share of their late husband's wealth. However, it changed with the Statute of Westminster II (1285) c. 34, when property holders may claim exceptions citing elopement and adultery to oppose widows’ attempts to claim their share [15].

Advocates and apologists for patriarchy have used the sexual division of labor to explain the gender roles propagated by patriarchy. According to Lerner, the progression of civilization from tribal existence into larger communities required different individuals to attend to varying necessary life activities [16]. The female was seen as the birthgiver and child-rearer, while the male was seen as the hunter, provider, and protector. The latter set of roles was perceived as the more highly valued one in society, which led to a sort of excuse for the legitimization of female inferiority. Apologists' views of gender continue to reference this idea of higher male value perpetuating male supremacy in society as an attempt to explain gender relations.

Marxism and Feminist Marxism

In the 19th-century movement of ideas of an egalitarian state with classless societies, Marxism completely rejects theological hegemony with its theories about gender and equality. Nonetheless, Friedrich Engels perpetuates an argument rooted in the hunter-gatherer society. Engels states that in such a society, women were demoted to the subordinate position of childbearer, caretaker, and provider of erotic pleasure to men. Paralleling Marxism, he maintains that the development of private property led to the "enslavement" of women [17]. This perspective draws from an evolutionary theory of sociobiology, however, a theory that has been largely debunked [18]. Feminist Marxism understands patriarchy as the capitalist mode of production. According to Sylvia Walby, patriarchy is "a system of social structures, and practices in which men dominate, oppress and exploit women" [19]. Juliet Mitchell refers to patriarchy as a system of kinship among men based on the commodification and exchange of women [20]. Eisenstein connects patriarchy to a sexual hierarchy, with the woman’s role relegated to domestic labor and procreator [21]. Patriarchy is thus often seen through a wider lens, including not only capitalism but also colonialism and racism. It should consequently be noted that in early Anglo-American feminist movements, women carried banners for not only women’s suffrage but also the abolition of slavery and ending the exploitation of children in the labor force. Furthermore, in the 1960s, women were championing not only feminism but also civil rights, an end to the Vietnam War, children’s welfare, and social and educational reform for individuals with disabilities [22]. The history of these opportunities for women is not a linear one but rather a 'snakes and ladders' type of projection in which the trajectory forward has often been plagued by societal failures and political pitfalls.

Anthropological and developmental points of view

Charles Darwin's The Descent of Man, and Selection in Relation to Sex argues that mate choice is explicitly aesthetic. Darwin repeatedly writes of mating preferences as an ‘aesthetic faculty’ and describes them as a "taste for the beautiful". These theories remain deeply problematic for millennials, whose reproductive options, sexual orientations, and gender identities have become more fluid. Contraception has revolutionized reproduction [23]. It has afforded innumerable individuals autonomy over their bodies. According to the natural selection argument that fitness and worth are intertwined with reproductive capability, contraception diminishes the worth of the person using it. Currently, with mounting political opposition to abortion and contraceptive rights, patriarchal factions seem bent on imposing laws on the bodies of women-another plummet down the game board of 'snakes and ladders.' These theories have lent themselves to arguments invalidating those who identify as part of the lesbian, gay, bisexual, transgender, queer (LGBTQ) community and/or undergo gender affirmation surgery.

Among mammals, sex differences in behaviors stem from differences in how females and males reproduce. According to Trivers, reproduction is an intensely time- and energy-consuming process for the female, beginning with gestation and continuing after birth to lactation and childrearing. For the male, however, there is less commitment, according to Trivers, in terms of time and effort, as the male commitment ends with fertilization [24]. Thus, Trivers refers to the interest of the male as simply maximizing the number of fertilized eggs to increase the chances of having children [24]. This divergence of interest, translated into human world-building based on ownership of private property and disproportionate valuation of male-male community bonds, and the resulting aggression can be seen as the beginning of the patriarchal domination of the female. In this view, females have been forced to surrender power and property to males to ensure heightened safety for their offspring, usually to the detriment of female advantages [25]. However, the anthropological account does not suggest that counterstrategies are entirely unavailable to females. Smuts found that after studying great apes, aggressive male sexual conquest of females was mitigated by social support from same-sex communalism among females [25]. The groundbreaking work of Margaret Mead laid the foundation for challenging gender roles and social norms around sexuality [26].

Perpetuation of systemic patriarchy

According to Walby, six structures-broadly termed the patriarchal mode of production, patriarchal relations in paid work, patriarchal relations in the state, male violence, patriarchal relations in sexuality, and patriarchal relations in cultural institutions such as religion, media, and education-perpetuate systemic patriarchy [19]. While these structures can be seen in the larger society, they are also seen in the familial unit, in which patriarchal tradition, practices, and ideals are vertically transmitted from generation to generation [27].

Parental guidance is shaped by patriarchal beliefs about gender norms, which are usually a large part of patriarchy in practice and perpetuated through households both consciously and unconsciously. The be-all and end-all goals of patriarchal practices remain control over female reproduction, and the ultimate sanction to achieve this goal is a violation of the basic human rights of others. Evolutionary analysis suggests that whenever we consider any aspect of gender inequality, we need to ask how it affects female sexuality and reproduction in ways that benefit men at the expense of women (and other men).

Anecdotal narratives of such indoctrination and subsequent commodification are rampant among women from the Indian subcontinent. It is not uncommon for families to be unwilling to send women to school, preferring to save up for their wedding expenses instead [28]. The culture of the extravagant wedding with the expectation of the bride’s family bearing the expenses underscores the pervasiveness of these practices. The oppression is often at a subversive level, enmeshed within the culture. Innocuous comments underlie the instinctive disdain for anything feminine, indicating that an achievement worth celebrating can only be achieved via a man. This has a deeply traumatic effect on the psyche of women, who learn to view themselves as inherently "less."

Parenting plays a formative role in the indoctrination of gender roles from infancy [29]. Parents make lasting decisions regarding a person’s gender identity from the time of birth, dictating the person’s name, pronouns, semantics, and activities. Any male child showing an instinctive preference for so-called feminine toys or colors may be chastised and ridiculed. Young girls may be encouraged to act in "womanly" ways, indicating a submissive and yielding attitude. This indoctrination of societal norms creates an oppressive environment, damaging the self-confidence of men and women.

Closely related to this is the issue of body image. Increasingly, cases of extreme anorexia and bulimia, often significantly heightened through exposure to a highly patriarchal social media feed, are becoming common among teenage girls and young women. The patriarchal convention of the "perfect woman," accompanied by a punishingly harsh physical model, bears a negative effect on impressionable psyches. Women are driven to desperate measures in their attempts to conform to unrealistic physical expectations. These psychological issues lead to intense physical harm and can even prove fatal in extreme cases. In a patriarchal society, gender identity is viewed through a fundamentally rigid heteronormative lens. The heteronormative standards are maintained as "normal," and as a result, any deviation from heteronormative behavior is presumed to be a form of deviance that needs treatment [30]. This has led to the labeling of homosexual and transgender individuals as diseased people who are then shunned and mistreated. Members of the lesbian, gay, bisexual, transgender, intersex, queer/questioning, asexual (LGBTQIA+) community face harassment and violence in society as their authentic gender identities lead to ostracism. Thus, oppressive social norms are linked to mental health disorders. 

The patriarchal system perpetuates a narrow, heteronormative, and archaic worldview. In the context of South Asian societies, certain patriarchal beliefs and practices can be traced back to history.

Patriarchy and its historical relationship with psychopathology

Malleus Maleficarum (Latin: Hammer of Witches), a detailed legal and theological document written in 1486 by Heinrich Kramer, an Inquisitor of the Catholic Church, was regarded as the standard handbook on witchcraft, including its detection and its extirpation, until well into the 18th century. Published 30 times between 1486 and 1669 and a best seller in Germany and France, it is a deeply disturbing misogynistic treatise on female religious transgression [31]. The Hammer of Witches is an especially egregious example, but in other historical periods too, we can discern societal developments that were particularly influenced by a patriarchal understanding of gender rules and behaviors.

Elaine Showalter explores this theme in The Female Malady, in which she discusses the development of psychiatry in England. The Victorian period, spanning almost the entire 19th century, was known for its rigid rules of conduct based on the division of the sexes [32]. It was also a time of enormous scientific discovery; studying the human mind became particularly exciting, and there was significant interest in understanding and treating mental ailments. Despite this rising interest in mental illness, the study was steeped in socially prevalent patriarchal beliefs, resulting in deep-rooted biases against women. Unsurprisingly, such institutionalized patriarchy, with its inherent misogyny, had a profound impact on the female psyche.

Numerous ailments were viewed as "feminine problems" and allotted a place of derision. This very naming of certain maladies brings this inbuilt bias to light. A psychiatric diagnosis of "hysteria" was often imposed on women suffering from epileptic fits, and the term is derived from the Latin term for "uterus." Similarly, "madness" was viewed as a feminine affliction in Victorian England, and treatments often included surgically removing internal female reproductive organs (The Hysterical Female). Psychiatrists often diagnosed perfectly sane women with insanity if they did not conform to social norms and conventions. Such diagnoses were often followed by incarceration in mental asylums with heinous practices including electric shock and lobotomies. Women, children, and the severely mentally ill were particularly likely to be lobotomized without their consent or, sometimes, even knowledge [33].

Generations of the scientific community theorized and perpetuated oppressive norms for women. In the last three decades of the 19th century, lobotomies gained widespread popularity for not only dysmenorrhea and ovarian neuralgia but also epilepsy, nymphomania, and insanity. Thousands of primarily young women had their healthy ovaries removed to cure them of a range of mental disorders that were believed to be caused by menstrual disorders. Thus, female sexuality, viewed as a dangerous aspect of femininity that needs to be kept under tight control, was increasingly viewed through a pathological lens [30]. Ironically, "madness," unnatural behavior, and trauma were often actually the results of women’s desperate efforts to live up to stifling social norms of conduct.

Sociologist Thomas J. Scheff referred to the relationship between power hierarchies in society and the labeling of individuals as mentally ill in Being Mentally Ill: A Sociological Study [34]. Socially negotiated power dynamics under the patriarchal system put men at the highest level of authority, and social-behavioral norms parallel patriarchal rules. Actions or behaviors that might threaten such social norms and conventions are summarily termed "deviant." Any behavior that could be related to mental illness was seen as deviant behavior, and thus, women who were relatively powerless in society became more susceptible to being labeled mentally ill [34].

Phyllis Chesler, in her 1972 book Women and Madness, argues that one of the largest causes of a numerically higher prevalence of women in mental patient populations is that "women, by definition, are viewed as psychiatrically impaired-whether they accept or reject the female role-simply because they are women" [30]. Women’s behavior is then devalued and even pathologized. Given that the world of psychiatry has also traditionally been overwhelmingly male, it is hardly surprising that patriarchal stereotypes of acceptable sex roles and the presumed inferiority of feminine traits underlie attempts to address mental ailments. Notably, it is because of the overwhelming presence of male thinking and the establishment of the study of the mind as a masculine enterprise that psychoanalysis continues to be more successful in understanding men than women.

Sigmund Freud (1856-1939) claimed that anatomy is destiny and that one’s gender determines one’s main personality traits (1973). This belief has continued to play a dangerously significant role in shaping how women are treated by men and even fellow women. Women have been taught that the fluctuations of their natural biology, such as menstruation, menopause, and pregnancy, are pathological conditions that incapacitate their ability to function [35]. While considering herself a disciple of Freud, Karen Horney (1995-1952) disagreed. She argued that the overwhelming impact of culture over biology was the primary determinant of personality. She refuted Freud’s claims that a woman’s sense of inferiority to the male sex stemmed from some universal process, what Freud referred to as "penis envy." She wrote, "[t]he wishes to be a man... may be the expression of a wish for all those qualities or privileges which in our culture are regarded as masculine, such as strength, courage, independence, success, sexual freedom, right to choose a partner" [36].

Developmental psychopathology and patriarchy

The societal impact of patriarchal attitudes toward women’s anatomy is nearly universal. Women are taught about the role and behaviors expected of them from infancy. The impact of patriarchal oppression on women can be found in presumably healing scenarios. In analytical psychology, the term "father complex" was developed both by Freud and Jung, and it's applied to a group of unconscious associations specifically about the image or archetype of the father [37]. Freud described the male child’s ambivalence toward parental authority in his multiple writings (*Rat Man* in 1909, *The Schreber Case* in 1911, *Totem and Taboo* in 1912, and *The Future of an Illusion* in 1927, etc.), as that which manifested as fear, defiance, and disbelief of the father, which could be interpreted as resistance to treatment. On the contrary, the Jungian view incorporates both males and females into the purview of the father complex. It theorizes that while a positive father complex is attributed to conformity with authority, a negative father complex could dispose one to an internalized image of all men as harsh, uncooperative, dominating, etc. [38].

In Western culture, the 1960s sexual revolution and subsequent feminist movements have had a huge impact on somewhat diminishing the gender gap [39]. However, in the non-Western world, including populous Southeast Asia, women continue to be subjected to violence in many forms, including domestic violence, rape, harmful traditional and customary practices, "honor killing," and trafficking [40]. In India, for example, it is common for young women to be treated as secondary to their male siblings. The needs of the male children in the patriarchal family are given precedence in aspects ranging from clothing and nourishment to education and medical attention. The idea that a woman is someone else’s property gets reemphasized at every step of her life. These beliefs go on to feed the unwillingness of parents to spend on their daughters' education. They attempt to justify this by stating that since the girl will have to be married off at a fairly early age, any benefits from her education will not accrue to the birth family and is thus seen as a waste of limited resources [41]. 

Scholars have observed the underlying phenomenon of commodification and "exchange of women," a socially accepted form of conduct that dehumanizes women and makes them a commodity to serve male requirements. Interestingly, this phenomenon also indicates a degree of male-male cooperation in humans that remains highly unusual in other mammals [42,43]. Negotiated marriages, bride stealing, and ritual defloration are common representations of this commodification. Women are indoctrinated from childhood to accept their subordinate roles and their obligation to their kin to accept such exchanges.

Impact of patriarchy on mental health

The patriarchal division of gender norms has set certain behavioral expectations for individuals based on their biological sex. Patriarchy perpetuates the psyche of equating biological sex with the socially constructed element of gender-pressurizing individuals in society to adhere to a strict set of narrow "acceptable behaviors" for each biological sex. This could be traumatic for individuals who may not necessarily want to adhere to such behaviors or extremely limiting gender boundaries. These individuals face alienation and ostracization and are more susceptible to sexual violence. For example, individuals from the LGBTQIA+ communities are frequently subjected to harassment and sexual abuse.

Thus, a patriarchal society makes for a fundamentally unsafe and detrimental space for non-conforming women and those who do not fit within narrow societal limits of gender and sexuality. Though this power imbalance may often be seen to exclusively benefit men, it has insidious dangers for their psychological well-being as well.

The rigid patriarchal outlook became particularly prominent in the social norms of the 19th century. William Alcott’s The Young Woman’s Book of Health (1850) and Edward H. Clarke’s Sex in Education or A Fair Chance for the Girls (1873) are both examples of instructive texts that were created based on the premise of female physical inferiority [44,45]. Needless to say, such a social outlook had a massive impact on the psychological well-being of women, who imbibed a predisposition towards submission based on presumed lower status, and thus accepted male aggression and violence towards them as normal and even necessary.

With the increasingly ubiquitous presence of social media, people are more vulnerable to patriarchy-induced deterioration of mental health. Social media and the internet have made it easier to perpetuate gender bigotry, support patriarchy, and spread negative portrayals of women [46]. Studies have specifically indicated that "social media use may be tied to negative mental health outcomes, including suicidality, loneliness, and decreased empathy" [47]. For example, social media platforms exhibit curated visual content promoting unrealistic lifestyles and body images that can trigger comparison, jealousy, and anxiety in individuals. Social media has also become a fertile ground for sexual predators [47]. Prepubescents and teenagers are particularly susceptible to falling victim to grooming, a practice in which an adult "builds a relationship, trust and emotional connection with a child or young person so they can manipulate, exploit and abuse them." Sexual violence is a major element of adverse childhood experiences (ACE) and leaves lifelong scars on a child's psyche. Feminist thinkers and intellectuals, beginning with Simone de Beauvoir, have postulated that a patriarchal society is built around and caters to male sexual instincts from early childhood [48]. Mary O’Brien has argued that male sexual violence is essentially a form of dominance display used to compensate for the male's inability to bear children [49]. According to Elizabeth Fisher, the mating practices and forced mating of animals became the source of inspiration for the human male to practice sexual violence [50]. This conducive social atmosphere gave root to men’s sexual dominance and institutionalized aggression.

In the 1970s, Susan Brownmiller, a member of the New York Radical Feminists, started a movement against the prevailing narratives around sexual violence. In a blistering rebuttal, she famously said, "rape is nothing more or less than a conscious process of intimidation by which all men keep all women in a state of fear." In 1975, her ground-breaking book Against Our Will was published, years after the foundational works of Kate Millett’s Sexual Politics and Shulamith Firestone’s The Dialectic of Sex [51].

Needless to say, sexual violence is experienced by both sexes, though the number of female victims significantly outnumbers male victims. Perpetrators, too, are overwhelmingly male, once again validating the fundamental definition of patriarchy as an institution of dominance aided by aggression and violence [52].

Adverse childhood experiences and subsequent trauma have life-altering impacts, often diminishing a person’s long-term well-being. A particularly brutal example of ACE is female genital mutilation (FGM)/cutting. This refers to the "surgical modification of the female genitalia, comprising all procedures involving partial or total removal of the external female genitalia or another injury to the female genital organs for cultural or nontherapeutic reasons" [53]. This practice continues to be prevalent in many parts of Africa and Asia, and sporadically around the globe. According to the World Health Organization, available data from 30 countries where FGM is practiced in the Western, Eastern, and North-Eastern regions of Africa and some countries in the Middle East and Asia reveal that more than 200 million girls and women alive today have been subjected to the practice, with more than 3 million girls estimated to be at risk of FGM annually. Victims of FGM often suffer prolonged health complications and even death, and survivors of the practice report extreme levels of trauma [54].

An oft-overlooked ill effect of patriarchy on mental health vis-à-vis gender is the negative impact it has on the well-being of men [55]. A large section of the male population faces incessant pressure to go against their natural inclinations and behave according to acceptable stereotypes. Men are expected to exude ‘masculinity’ in their everyday lives by negating emotions and adopting an aggressive attitude. Boys face ruthless bullying and cruelty from peers if they display sensitivity or other ‘feminine’ traits. As a result, they learn to suppress emotions and adopt a lifestyle that normalizes violence to live up to patriarchal gender constructs [56]. It has been long documented that men, on average, have a shorter lifespan than women. While some of this can be attributed to genetic and biological factors, it is also largely exacerbated by increased risk-taking behaviors and the consequent heightening of stress levels in men [57]. A large proportion of men exhibit signs of stunted emotional development, which eventually leads to difficulties in forming and maintaining relationships as adults. Stereotypes about male resilience and ‘toughness’ prevent men from seeking mental health help, which worsens an already difficult situation. There may be a hidden cost of patriarchy in the mounting burden of mental disorders, but it is yet to be estimated. However, inferences could be drawn from recommendations from gender equity commissions that, when adopted, have yielded improvements in economic indicators and workforce productivity after decades of advocacy for equal pay and labor opportunities. 

Addressing patriarchy in clinical encounters

It bears repeating that patriarchy is a fundamentally oppressive, all-pervasive system that permeates all aspects of life. The impact of this disruptive system is exemplified in the physical, emotional, financial, and socio-political realms, and as argued here, no more so than in the realm of mental health and disorder. As clinicians, we encounter persons with very different concerns. What can clinicians do?

We, as clinicians, are accustomed to making personal inquiries into all aspects of a person's life, particularly into their competencies founded upon their attachments, their adverse life events, and how these shape their embodied experience of current stressors. Further inquiries into their stress responses in the body and mind, coping strategies, and the experience of demoralization are distinct from but interactive with any psychopathological interferences there may be in their pursuit of personal flourishing. Life-affirming values provide a motive force for countering demoralization and trajectories toward harm and restoring them to their trajectory of personal flourishment. And lastly, their support systems provide scaffolding and guidance when individual efforts to retrieve life-affirming values falter.

Instead of a linear image of climbing ladders, scala in alignment with the ways of human flourishing is required, examples of which we have from antiquity and which can be conjured by clinicians from childhood memories. Such as the game of 'snakes and ladders' (aka chutes and ladders) that depicts roadblocks, setbacks, and effortful turnarounds. With this image in mind, if clinicians construe patriarchal oppression to be among the factors potentially affecting competencies, contributing to ongoing stressors while constraining adaptive stress responses, contributing to demoralization, affecting the formation or deformation of values supporting or undermining revaluations, factors lodged firmly among the encrypted structural biases that lead to social system failure, then clinicians can begin shaping clinical inquiries into the snakes and ladders board game in which persons have been given roles to play [58,59].

**Figure 2:**
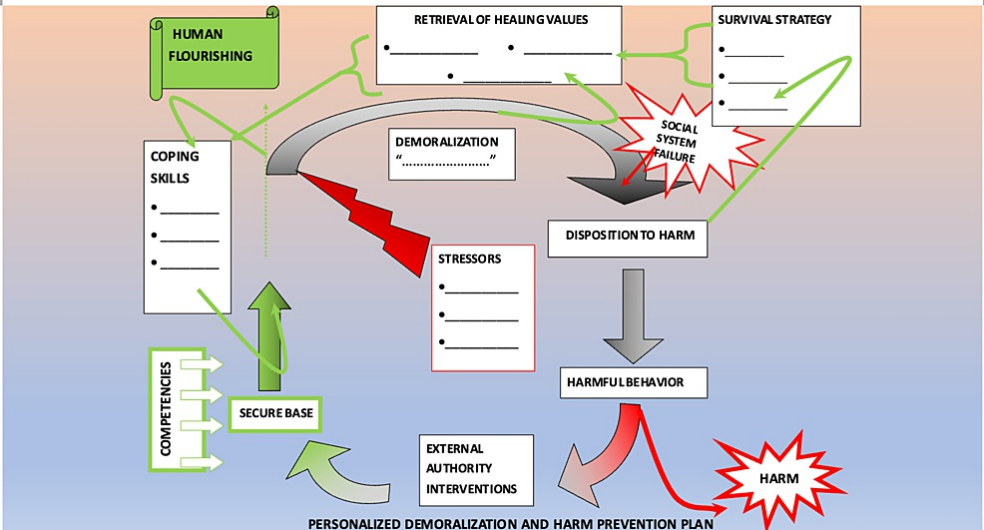
A brief overview of addressing patriarchy during clinical encounters Image used with permission from Dr. Matthew Galvin of the Indiana University Conscience Project.

Some people will have concerns about the role they perceive they have been assigned and seek to challenge it. Some will seem content with the rules as received, while others will discern the injustices inherent in them and call for new ones. Some will deeply wonder about the construction of the game board itself. In each case, the clinician has the privilege of making personal inquiries. See Figure 2 for details.

Limitations 

There are several limitations to this narrative review. The potential for selection bias, given that it is not based on protocol-based searches, provides a piece of weaker evidence. Given that patriarchy is not yet established as a valid mental health construct, there is a dearth of studies that measure its association with developmental psychopathologies. Also, this is a broad review of patriarchy and attends to multiple faculties within scientific disciplines to provide a critique of many pieces of information together in a readable format.

## Conclusions

The virtues that supported human evolution since the Neolithic ages are no longer of similar importance. Especially since inherent subjugation failed to stand the empirical verification process and societal perspectives began to alter towards progressiveness during the 14th-century Renaissance period.

Likewise, it is critical to validate the subjective experiences of those affected across genders and recognize and acknowledge the plausible negative effects of patriarchy on mental health. Several confounding variables require robust empirical scrutiny, and the crucial first step is to spread awareness regarding patriarchy. While the negative impact of patriarchal oppression on women and other minority communities has been long recognized across many disciplines, it is vital to highlight that the advantages of this institution for men are frequently overridden by severe detrimental and long-term deleterious effects. The recognition of this universal construct perpetuated by existing systems is imperative for institutional overhaul. It is undoubtedly a tricky proposition, as it will involve a paradigm shift in the societal power dynamics of gender and heteronormativity and will certainly encounter resistance from many quarters. Overhauling a systemic, institutionalized philosophy will involve identifying and eradicating the instruments that perpetuate patriarchy. The decades-long scholarship by feminist thinkers, mental-health experts, and social workers will be of immense value in this endeavor.

As John Stuart Mill pointed out in *The Subjection of Women* (1873), we cannot know the inherent nature of the sexes as long as we are reared in environments in which women are subordinate. Until gender equality exists, we cannot claim to know what shape the natural unfolding of male and female psyches will take. The experience of nearly gender-equitable societies such as those in Scandinavian nations indicates that a society free of patriarchal oppression leads to improved mental and physical health and a thriving and prosperous community.
